# Toxin constraint explains diet choice, survival and population dynamics in a molluscivore shorebird

**DOI:** 10.1098/rspb.2013.0861

**Published:** 2013-07-22

**Authors:** Jan A. van Gils, Matthijs van der Geest, Jutta Leyrer, Thomas Oudman, Tamar Lok, Jeroen Onrust, Jimmy de Fouw, Tjisse van der Heide, Piet J. van den Hout, Bernard Spaans, Anne Dekinga, Maarten Brugge, Theunis Piersma

**Affiliations:** 1 Department of Marine Ecology, Royal Netherlands Institute for Sea Research (NIOZ), PO Box 59, 1790 AB Den Burg (Texel), The Netherlands; 2 Animal Ecology Group, Centre for Ecological and Evolutionary Studies (CEES), University of Groningen, PO Box 11103, 9700 CC Groningen, The Netherlands; 3 Centre for Integrative Ecology, Deakin University, Waurn Ponds Campus, Geelong, Victoria 3217, Australia; 4 Community and Conservation Ecology Group, Centre for Ecological and Evolutionary Studies (CEES), University of Groningen, PO Box 11103, 9700 CC Groningen, The Netherlands

**Keywords:** diet choice, hydrogen sulphide, optimal foraging theory, predator–prey interactions, survival rate, toxins

## Abstract

Recent insights suggest that predators should include (mildly) toxic prey when non-toxic food is scarce. However, the assumption that toxic prey is energetically as profitable as non-toxic prey misses the possibility that non-toxic prey have other ways to avoid being eaten, such as the formation of an indigestible armature. In that case, predators face a trade-off between avoiding toxins and minimizing indigestible ballast intake. Here, we report on the trophic interactions between a shorebird (red knot, *Calidris canutus canutus*) and its two main bivalve prey, one being mildly toxic but easily digestible, and the other being non-toxic but harder to digest. A novel toxin-based optimal diet model is developed and tested against an existing one that ignores toxin constraints on the basis of data on prey abundance, diet choice, local survival and numbers of red knots at Banc d'Arguin (Mauritania) over 8 years. Observed diet and annual survival rates closely fit the predictions of the toxin-based model, with survival and population size being highest in years when the non-toxic prey is abundant. In the 6 of 8 years when the non-toxic prey is not abundant enough to satisfy the energy requirements, red knots must rely on the toxic alternative.

## 1. Introduction

Toxic food is better avoided, and there is a large literature on how predators learn to avoid toxic prey [1–6]. Nevertheless, an emerging alternative view is that predators should not entirely neglect toxic prey as long as this could increase their opportunity to gain energy [7–13]. Mildly toxic prey species that are not directly lethal upon ingestion could be valuable during times when non-toxic food is in short supply [14,15]. There are a number of cases where predators have been reported to consume toxic but not-immediately-lethal prey [16–21], but the dietary choices [7,17,19–21] and subsequent demographic consequences [18] remain unexplained in mechanistic and functional terms.

Optimization models may help us to understand how predators should strategically trade off the minimization of toxin ingestion with the maximization of energy gain. Recent state-dependent models predict that the hungrier a predator is, the more likely it is to accept toxic prey [9,10], a prediction that was upheld that empirically [8,13]. Furthermore, through a predator's hunger state, the willingness to include mildly toxic prey should depend on the abundance and availability of non-toxic food, which is a prediction that allows field testing. However, when it comes to field testing, in both the models and the experiments, the only difference between prey types was their degree of toxicity, and this may be quite unrealistic.

In nature, prey species differ in many more defence traits than degrees of toxicity. By making it difficult for a predator to detect, capture, ingest or digest prey [22,23], non-toxic and nutritious prey species may escape predation. Predators therefore need to deal with multiple constraints, and may face much steeper trade-offs between energy gain and toxin avoidance than hitherto assumed. Here, we will focus on such a system in which a predator faces the choice between an easy to digest toxic prey and a much harder to digest non-toxic prey. Building upon the existing digestive rate model (DRM) developed by Hirakawa [24], which includes a digestive constraint but not a toxin constraint, we have developed a novel toxin-digestive rate model (TDRM) to generate food-density-dependent predictions on optimal diet and maximum energy intake rates for systems where prey differ in toxicity. The predictions of both the DRM and the TDRM are then put to the test in an 8-year field study on food abundance, diet choice, survival rate and population size in a molluscivore vertebrate predator, the red knot (*Calidris canutus canutus*, hereafter knot), in its non-breeding area at Banc d'Arguin (Mauritania), characterized by a highly sulfidic environment in which the most abundant mollusc prey is toxic, while other prey types are not.

### (a) Study system

The intertidal flats at Banc d'Arguin are densely covered by seagrass (mainly *Zostera noltii* Hornem.) [25]. Detritus is produced at a high rate, which is degraded anaerobically by sulphate-reducing bacteria [26], causing a build-up of high concentrations of hydrogen sulphide in sediment pore water [27,28]. Sulphide is toxic to many organisms as its lipid solubility enables it to freely penetrate biological membranes, eventually slowing down the functioning of mitochondria and the production of ATP [29]. A specialized group of organisms that can profit from high sulphide concentrations in seagrass beds are Lucinidae [30], heterodont bivalves that live in symbiosis with chemoautotrophic bacteria inside their gill structures [31]. These bacteria oxidize sulphide that is provided by the lucinid host to synthesize sugars which fuel both the growth of the lucinid host and its endosymbiotic bacteria [32]. The lucinid *Loripes lucinalis* (hereafter *Loripes*) is the dominant bivalve in Banc d'Arguin, with densities of up to 4000 individuals per m^2^ [33,34], and hence Banc d'Arguin can be considered as a chemosynthesis-based ecosystem [35].

Banc d'Arguin is an important non-breeding area for Arctic-breeding shorebirds, hosting more than two million individuals in winter, with knots being the most abundant molluscivore [36]. Knots face a trade-off between feeding on the superabundant but toxic *Loripes* [37] and a much less abundant but non-toxic prey, *Dosinia isocardia* (hereafter *Dosinia*); numerically, *Loripes* and *Dosinia* together make up 75 per cent of all molluscs that are ingestible by knots [38,39] and dominate the diet of knots [40]. Knots face an additional trade-off: *Loripes* has a very thin shell, whereas *Dosinia* has a thicker armature. As knots ingest their prey whole [41], they often face a digestive processing constraint [42], which can be alleviated by selecting bivalves that have high flesh-to-shell mass ratios [43]. The toxicity of *Loripes* for knots has recently been investigated experimentally [37]. Captive knots that were given a *Loripes*-only diet quickly developed diarrhoea, thereby losing significant amounts of water. Their compensatory water consumption could not prevent a decrease in food intake. When given a diet of non-toxic *Dosinia*, birds recovered within an hour. Intake rates on *Loripes* available ad libitum were three times lower than expected on the basis of maximal shell mass processing rates, whereas intake rates on *Dosinia* available ad libitum matched the prediction of a model that predicted intake as constrained by the processing of shells. When given the choice between *Dosinia* and *Loripes*, the captive birds included both prey types in their diet, which maximized their energy intake rate as predicted by a model developed for ad libitum situations.

### (b) Toxin-digestive rate model

The TDRM is developed for non-ad libitum circumstances, where foragers need to search for their prey. In its most simple form, it assumes that there are just two prey types *i* = 1,2, which can each be characterized by energy contents *e_i_*, indigestible ballast mass *k_i_*, toxin contents *s_i_*, handling time *h_i_*, searching efficiency *a_i_* and density *D_i_*. The problem is finding the acceptance probabilities ***P*** = (*p*_1_, *p*_2_) for both prey types that maximize the forager's long-term energy intake rate *Y*. The latter is given by the multi-species version of Holling's disc equation [44]:1.1
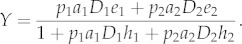
In the ‘classical prey model’ [45], which ignores possible digestive and toxin constraints, finding the optimal solution is straightforward. First, rank prey types such that 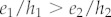. Always accept type 1 (*p*_1_ = 1), and accept type 2 (*p*_2_ = 1) whenever 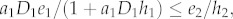 otherwise reject (*p*_2_ = 0). This model, called the ‘contingency model’ (CM) [46], has been upheld in many diet studies on a variety of foragers [47], but was refuted in the case of knots [43,48]. As knots face a digestive constraint, they should and do take a prey's ballast mass into account when selecting their diet [43].

**Figure 1.**
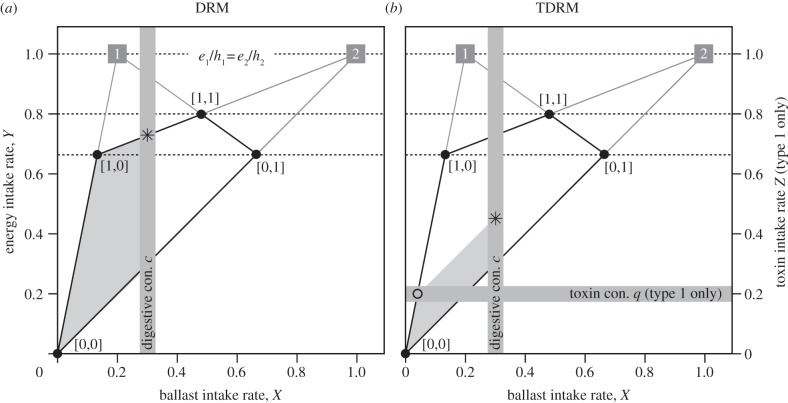
Graphical solution, following Hirakawa [24] and van Gils *et al.* [43], to find the optimal choice between two prey types, which maximizes energy intake rate (asterisk in both graphs) under (*a*) one or (*b*) two constraints. In both graphs, squared symbols give maximum intake rates at infinite densities of either type 1 or type 2 when there would be no constraints, kite-shaped surface bordered by black solid lines gives feasible intake rates under (given) finite prey densities, grey area within the kite shape gives feasible intake rates under the acknowledgement of (*a*) a digestive constraint (con.) and (*b*) both a digestive and a toxin constraint. Numbers in squared brackets give diet choice as [*p*_1_, *p*_2_]. (*a*) Accounting only for a digestive constraint, the DRM ranks prey types on the basis of digestive quality (*e*/*k*) and predicts for this case that the high-quality prey (type 1) should be fully accepted (*p*_1_ = 1), whereas the poor-quality prey (type 2) should only be partially selected (0 < *p*_2_ < 1). (*b*) Accounting for both constraints, the TDRM predicts partial preference on both prey types (0 < *p*_1_ < 1 and 0 < *p*_2_ < 1). Maximum energy intake rate is found by drawing a line parallel to the lower line of the kite shape (this line is parallel because toxin intake rate *Z* is kept at *q* across this line), starting where toxin constraint *q* crosses the left-most line of the kite shape (open dot) until it hits digestive constraint *c* (asterisk). Note that the scenario plotted here mimics our study qualitatively (the only toxic prey is the type with the highest *e/k* ratio), but not quantitatively (parameter values have been chosen arbitrarily).

If ballast intake rate *X* for the optimal solution in the CM exceeds digestive constraint *c*, then the forager faces a digestive bottleneck, in which case the CM yields a suboptimal solution [24]. Then, the rate-maximizing diet choice can be found using the DRM [24]. This model can be solved graphically by plotting energy intake rate *Y* against ballast intake rate *X* for all possible combinations of ***P***, including partial preferences for either type (figure 1*a*). Then, by drawing digestive constraint *c* (vertical bar in figure 1*a*), one can work out which diet choice ***P*** yields the maximum sustainable energy intake rate *Y* under constraint *c* (asterisk in figure 1*a*). For details, we refer to the original paper by Hirakawa [24] and its first applications in knots [43], for which such an ‘all-or-nothing constraint’ has explained intake rate [42], prey choice [43,48], patch choice [49], selection of stopover sites [50] and even digestive organ sizes [42,51,52]. As already mentioned by Hirakawa [24], the same graphical procedure can be followed when the forager faces a toxin *rather than* a digestive constraint (replacing ballast intake rate *X* by toxin intake rate *Z* and ballast contents *k_i_* by toxin contents *s_i_*).

However, a forager's energy intake rate may be bottlenecked by *both* a digestive and toxin constraint. This occurs when, accounting for digestive constraint *c* in the DRM (i.e. when *X* > *c* in the optimal CM solution), toxin intake rate *Z* in the optimal DRM solution exceeds *q*. This can occur only when the highest-digestive-quality prey (i.e. the one with the highest *e_i_*/*k_i_*) is most toxic (i.e. the one with the highest *e_i_*/*s_i_*; in our *Loripes*–*Dosinia* case, this condition was always upheld; electronic supplementary material, table S1). Graphically, the optimal solution under both constraints can be found by adding a third axis to Hirakawa's state space (figure 1*b*; note that we have added the third axis to the existing two-dimensional plane, making reading the details easier; we could have also plotted *X*, *Y* and *Z* three-dimensionally). Solving the model analytically is equally straightforward and we will refer to it as TDRM (note that TDRM equals a DRM when only one of the two constraints operates, which in turn equals a CM when none of the constraints is present). First, maximal sustainable ballast intake rate *X* is set by digestive constraint *c*,1.2a
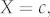
which can be written as1.2b
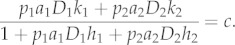
Similarly, maximally tolerable toxin intake rate *Z* is set by toxin constraint *q*,1.3a
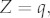
which can be written as1.3b
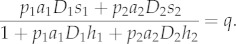
Solving equations (1.2*b*) and (1.3*b*) for the two unknown variables *p*_1_ and *p*_2_ yields the optimal acceptance probabilities1.4
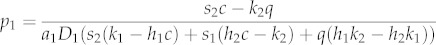
and1.5
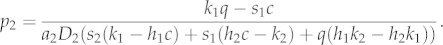


## 2. Material and methods

### (a) Benthos

Our study period spans from 2003 to 2010, in which we collected 1024 benthos samples in 13 consecutive expeditions: Dec. 2003 (*n* = 84), Dec. 2004 (*n* = 26), Apr. 2005 (*n* = 39), Dec. 2005 (*n* = 8), Nov. 2006 (*n* = 6), Apr. 2007 (*n* = 229), Aug. 2007 (*n* = 8), Oct. 2007 (*n* = 12), Feb. 2008 (*n* = 142), Apr. 2008 (*n* = 78), Nov. 2008 (*n* = 56), Oct. 2009 (*n* = 224) and Oct. 2010 (*n* = 112). Following procedures described elsewhere [28,43,49], a benthos sample represented a sediment core (diameter: 15 cm) taken to a depth of 20 cm and sieved over a 1 mm sieve. Top (0–4 cm) and bottom (4–16 cm) parts of the sample were sieved separately in order to distinguish between prey that are accessible and inaccessible to knots [53]. In the laboratory, each mollusc was identified to species level, and shell length was determined (± 0.1 mm). The latter allowed us to distinguish between ingestible and non-ingestible prey (knots can ingest all size classes of *Loripes* and *Dosinia* < 13.2 mm). By drying (3 days at 60°C), weighing (±0.1 mg) and incinerating (5 h at 550°C) flesh and shell separately, we determined individual flesh ash-free dry mass AFDM_flesh_ and shell dry mass DM_shell_ from subsamples. The relationships of AFDM_flesh_ and DM_shell_ with shell length were used to predict missing values for those prey items that were not weighed. Next, numerical density (*D* in equations (1.1)–(1.5)), AFDM_flesh_ (*e* in equation (1.1)) and DM_shell_ (*k* in equations (1.2*b*), (1.4), (1.5)) were averaged per year per species (available items only, i.e. those accessible and ingestible), diet models and were used to calculate available biomass densities and as input variables in the two diet models (see the electronic supplementary material, table S1; toxin contents *s* was equated to flesh contents *e* in case of *Loripes* because toxin constraint *q* is expressed in terms of *Loripes* flesh intake). Further parameter values used were searching efficiency *a* = 4 cm^2^ s^−1^ [28,54], handling time *h* = 1 s [28], toxin constraint *q* = 0.1 mg AFDM_flesh_ s^−1^ [37] (*Loripes* only) and gizzard mass = 10 g [50], resulting in digestive constraint *c* = 5 mg DM_shell_ s^−1^ [42].

All samples were taken in the vicinity (less than 5 km) of Iwik, Banc d'Arguin (19°53′ N, 16°18′ W). Samples collected in 2003, 2004 and 2006 were taken closer to Iwik (0–3 km) than in other years (1–5 km). Spatial differences at this scale might have had little influence. Yet smaller-scale spatial parameters such as distance to gullies, affecting the presence of seagrass [55], might have had a larger effect. *Loripes* is mostly found in seagrass, whereas *Dosinia* is almost as abundant in bare as in seagrass habitat [38], and differences in prey densities between years may thus in part be due to differences in spatial design (on average, seagrass covers 80% of the intertidal surface at Banc d'Arguin [25]). We tested potential biases for both spatial scales by comparing our 2004 data (0–3 km to Iwik) with those of an independent study also from 2004 by Honkoop *et al*. [38], who sampled mudflats 1–5 km away from Iwik and took an equal number of samples in bare and in seagrass habitat. 2004 was a notable year in which *Dosinia* was more abundant than *Loripes* (1142.7 versus 23.9 m^−2^ in our study and 216.6 versus 198.2 m^−2^ in the study by Honkoop *et al*. [38]; after correcting their stratified data for the 80% seagrass coverage of the intertidal flats and for the species-specific availability fractions, 0.73 for *Dosinia* and 0.70 for *Loripes* [28]). We repeated all analyses by replacing our 2004 benthos data by those of Honkoop *et al*. [38], which revealed that neither the outcome of the survival analyses nor the outcome of the diet comparisons was sensitive to our spatially inconsistent sampling programme (see the section on sensitivity analysis with respect to benthos sampling in the electronic supplementary material).

### (b) Diet composition

During six of the 13 expeditions, we collected 77 faecal samples (2003, *n* = 21; 2004, *n* = 6; Apr. 2007 *n* = 8; Oct. 2007, *n* = 14; Feb. 2008, *n* = 11; 2009, *n* = 17), samples usually containing 40–60 droppings. Samples were sorted using standard methodology [56], which has recently been calibrated for knots feeding on *Dosinia* and *Loripes* [40]. In short, after drying (3 days at 60°C), shell fragments that were retained on a 300 μm sieve were sorted out and weighed per species, yielding species-specific estimates of ingested DM_shell_ (after correcting for 35% of DM_shell_ not being retained on the sieve [40]). Next, hinges were assorted to species and their heights were determined in order to reconstruct ingested size distributions. The latter was needed to express a species's relative diet contribution in terms of total AFDM_flesh_ consumed, because AFDM_flesh_/DM_shell_ ratios are size-dependent [56]. Relative diet compositions were logit-transformed before calculating the annual averages [57].

### (c) Annual survival rates

Survival estimates were based on capture/resighting data of a total of 1595 individually marked knots. The birds were captured and resighted during annual three-week expeditions in November/December 2002–2010 [58], yielding annual survival estimates for seven consecutive years (2003–2009; because survival rate cannot be separated from resighting probability for 2010 when modelled with time dependence). The birds were aged upon capture [59], distinguishing hatch-year birds (juveniles) from older birds (adults). Apparent (or local) survival (*Φ*) and recapture probabilities (*p*) were estimated from live encounter data using Cormack–Jolly–Seber models [60]. As benthos and diet data were collected throughout the entire study area, we pooled the data of the two sites in our study area, Abelgh Eiznaya and Baie d'Aouatif [58,61]. Based on knowledge gained from earlier analyses, we made some *a priori* assumptions to reduce the number of parameters in order to increase the precision of the survival estimates: it has been shown that a time-since-marking (tsm) effect explained most of the variation in annual survival [58], and we thus considered tsm effects to account for transients or handling effects on survival in the first year after capture (*Φ*^1^) versus subsequent years (*Φ*^2+^). It has further been shown that age at capture (adult versus juveniles) explained a significant part of the variation in survival [58], and we thus included age at capture in our models. Note that knots were treated as adults after their first year (more than 12 months of age), and consequently no age differences existed within the *Φ*^2+^ category. As we were interested in which of the two diet models best explained the annual variation in survival rate, we included intake rates predicted by the TDRM and DRM, respectively, as continuous variables in the models. Additionally, to test for survival differences among years, we included time as a factor (time), but also tested whether there was a linear trend in survival rate over time (Time), because an earlier analysis indicated a decline in knot survival over time [62]. In all models, resighting probability *p* was modelled as a function of time (again as a factor) and site, as observation effort differed between the two sites, and logistic improvements suggested resighting efforts differed between years [58]. Both adults and juveniles forage on open mudflats during low tide and assemble at roosts during high tide, and we had no reason to expect *p* to differ between age classes.

The global model was *Φ*_age × tsm__+__time_ *p*_site__+__time_ and we tested the goodness of fit using the median-ĉ (c-hat) test implemented in the mark software v. 6.0 [63]. The level of overdispersion was estimated at *ĉ* = 1.05 ± 0. Models were constructed and run in R (v. 2.15.0) using the RMark v. 2.1.4 package [64] as an interface for program mark [63]. We used model averaging to calculate survival and resighting probability, and present parameter estimates as 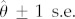 Model selection was based on Akaike's information criterion corrected for small sample size and overdispersion (*ĉ*; QAIC_c_). Based on the earlier-mentioned assumptions, the candidate model set consisted of all biologically and ecologically plausible combinations of parametrizations for *Φ* and *p* (see the electronic supplementary material, table S2).

### (d) Estimating and predicting population dynamics

Each year between 2002 and 2010, we carried out a single count of all knots roosting in the Iwik study region. This took place during a daytime spring high tide in November/December. Birds were counted using telescopes by four or five teams of two observers, each counting a subsection of our study area.

We modelled the population trend for 2002–2010 using adult and juvenile survival rates estimated by the most parsimonious model (i.e. survival model 1 in electronic supplementary material, table S2). In this statistical model, TDRM energy intake rates *Y* served as input, which were predicted on the basis of equation (1.1) using (i) the observed densities of both *Loripes* and *Dosinia*, (ii) the observed densities of *Loripes* only, and (iii) the observed densities of *Dosinia* only. The last two hypothetical scenarios allow us to hypothesize how much knot population dynamics depend on the presence of either *Loripes* or *Dosinia*. As applied before when modelling knot population dynamics [65], we used a two-dimensional matrix population model, in which fecundity (*f*; equal to 0 for juveniles and 0.14 yr^−1^ for adults [66]), juvenile survival (*Φ*_juv_) and adult survival (*Φ*_ad_) determine how the number of juveniles (*N*_juv_) and adults (*N*_ad_) in year *t* affect the number of juveniles and adults in year *t* + 1:2.1
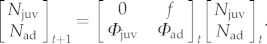
The 2002 count was used as the initial population size in the model.

## 3. Results

### (a) Annual survival rate

**Figure 2.**
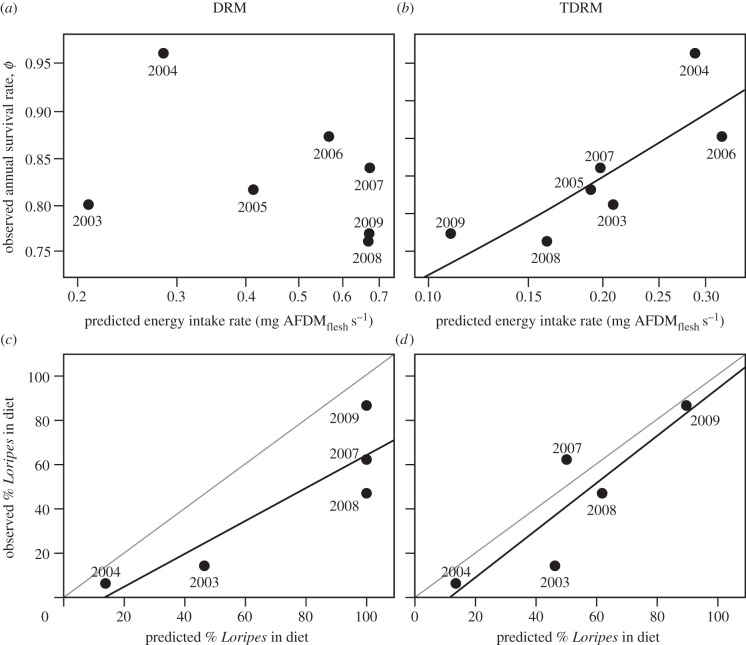
(*a*) Year-specific adult survival rate (estimated by model 2 in electronic supplementary material, table S2; year runs from Nov/Dec of the previous year to Nov/Dec of the plotted year) does not correlate with the DRM-predicted intake rate, (*b*) whereas it correlates positively with the intake rate predicted by the TDRM. Line gives model fit (model 1 in electronic supplementary material, table S2). (*c*) Observed amounts of *Loripes* in the diet (relative to *Dosinia*) are lower than predicted by the DRM, (*d*) but match with TDRM-predictions. Grey lines represent *y* = *x* lines, and black lines are significant regression lines.

TDRM models were substantially better supported than models including DRM intake rates (cumulative QAIC_c_ weight: 0.38 for models including TDRM intake rates, and 0.00 for models including DRM intake rates; electronic supplementary material, table S2; figure 2*a,b*). Although models including annual variation as explanatory factor (i.e. factor time) scored high in the model selection process (cumulative QAIC_c_ weight: 0.48; electronic supplementary material, table S2), they added extra parameters (complexity) to the models and should thus be less favoured. There was no evidence for a time trend in survival (i.e. models including Time; cumulative QAIC_c_ weight: 0.14; electronic supplementary material, table S2). Furthermore, there was no support for adult survival being different in the first year after marking, compared with subsequent years (model 2 versus model 3, *Δ*QAIC_c_ = 0.34). Model-averaged survival estimates can be found in the electronic supplementary material, table S3.

### (b) Diet composition

The observed contribution of *Loripes* to the diet was less than predicted by the DRM (figure 2*c*; *t* = −3.44, d.f. = 4, *p* = 0.03). For 3 of 5 years for which we had diet data available, the DRM predicted that knots should fully ignore *Dosinia* (figure 2*c*). In those three years (2007, 2008, 2009), the abundance of *Loripes* was so high that, even if knots would feed on *Loripes* only—the prey with the highest flesh-to-shell mass ratio—their gizzard would not be able to achieve the required shell mass processing rate (i.e. knots would face a digestive constraint). Hence, only a proportion of encountered *Loripes* should have been accepted (see the electronic supplementary material, table S4; note that this is different from conceptual figure 1*a* where, for reasons of visual clarity, we assumed that even maximum ballast intake rates on prey type 1, i.e. *k*_1_/*h*_1_, are below digestive constraint *c*).

By contrast, diet compositions predicted by TDRM matched the observed diets (figure 2*d*; *t* = −1.26, d.f. = 4, *p* = 0.28). In 3 of 5 years, the intake rate on *Loripes* would have exceeded the toxin constraint if all encountered *Loripes* were accepted. Hence, only a proportion of the encountered *Loripes* should have been accepted for this reason (see the electronic supplementary material, table S4). In those years, knots following the TDRM could accept all encountered (ingestible) *Dosinia* as the occurrence of the toxin constraint kept shell mass processing rates low, and thereby prevented a digestive constraint. Only in the year that *Loripes* was less abundant than *Dosinia* (2004) does the TDRM predict a digestive rather than a toxin constraint. In 2004, knots should thus have accepted all encountered *Loripes* and only a fraction of the encountered (ingestible) *Dosinia* (see the electronic supplementary material, table S4).

### (c) Predicted and observed population dynamics

**Figure 3.**
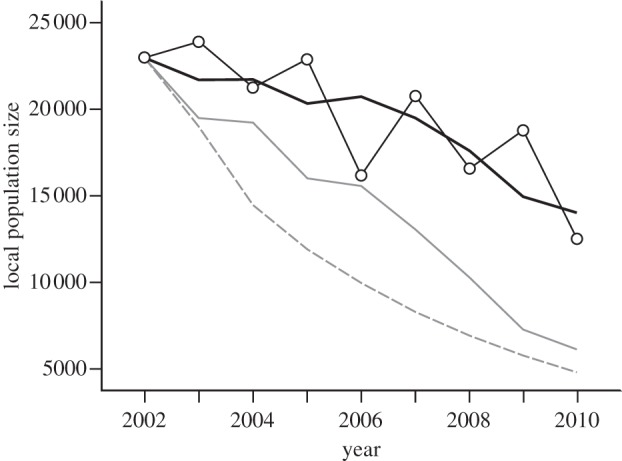
Predicted population dynamics of knots in the presence of both prey (thick black line), in the absence of *Loripes* (solid grey line) and in the absence of *Dosinia* (dashed grey line). Observed population size (circles connected by thin black line) follows predicted population decline based on both prey.

Predicted knot population size declined over time, with the decline being steepest if *Dosinia* would have been removed from the system (−79% from 2002 to 2010), followed by the scenario when *Loripes* would have been removed (−74%). However, even with both prey included in the diet, knot numbers were predicted to decrease over time (−39%; figure 3). This last model agreed best with the observed decline in knot numbers from 22 859 in 2002 to 12 465 in 2010 (−45%; figure 3).

## 4. Discussion

**Figure 4.**
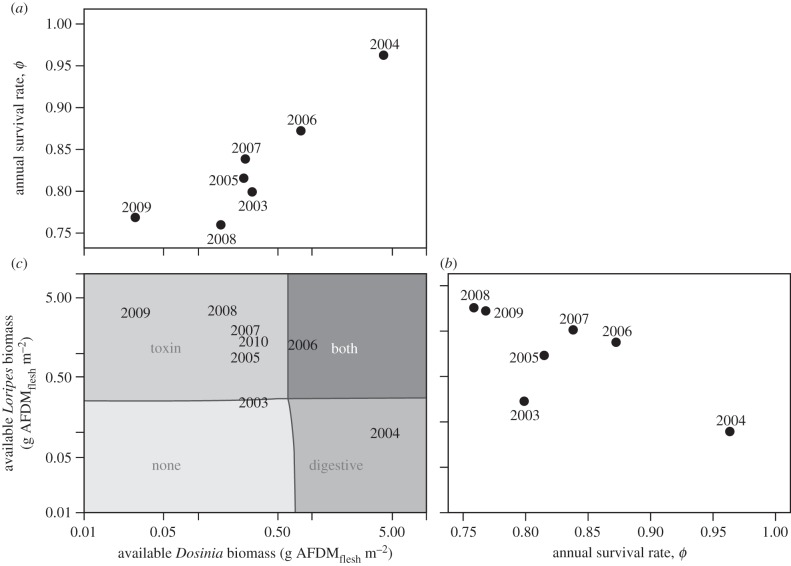
(*a*) Year-specific adult survival rate (estimated by model 2 in the electronic supplementary material, table S2) correlates with the available biomass density of *Dosinia*, (*b*) but not with *Loripes* density. (*c*) These prey densities themselves correlate negatively. Grey shading in the background indicates whether knots would face a toxin constraint, a digestive constraint, both constraints or neither. For details behind these calculations, see the electronic supplementary material, ‘figure 4*c* explained’.

Knot annual survival rates correlated strongly with annual variations in *Dosinia* abundance (figure 4*a*; Pearson's *r* = 0.91), but showed no trend with *Loripes* abundance (figure 4*b*; Pearson's *r* = −0.72). This strongly suggests that knots need non-toxic *Dosinia* to survive and cannot rely on *Loripes* only, even though *Loripes* is much more abundant and has a much higher flesh-to-shell ratio. The reasoning for this dependency is rather simple: in order to prevent lethal intoxication, knots can ingest *Loripes* up to a rate that is only half of their required intake rate [37], and they need prey such as *Dosinia* to meet their energy demands. On the other hand, *Dosinia* was not abundant enough for knots to fully rely on them as an energy source.

**Figure 5.**
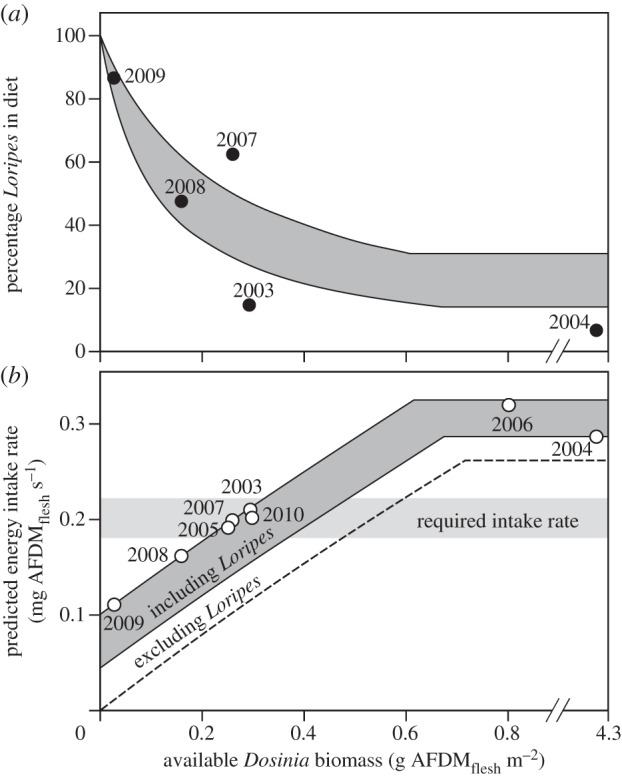
(*a*) How the amount of *Loripes* in the diet (relative to *Dosinia*) relates to the available density of *Dosinia*, both theoretically (TDRM) and empirically. Theoretical predictions are given by the grey band, with lower line representing a poor *Loripes* density (0.1 g AFDM m^−2^) and upper line a higher *Loripes* density (≥0.25 g AFDM m^−2^; as knots face a toxin constraint at *Loripes* densities of at least 0.25 g m^−2^, diet composition becomes independent of *Loripes* abundance above such densities). Diet composition becomes independent of *Dosinia* density when the digestive constraint is met (i.e. above *Dosinia* densities of 0.6–0.7 g m^−2^). (*b*) TDRM functional response to variations in *Dosinia* density. Grey band as in (*a*) shows that most variation in intake rate is due to density variations in *Dosinia* rather than in *Loripes*. Nevertheless, without *Loripes*, intake rates would be substantially lower (dashed line) and often below the level required for subsistence. Dots denote year-specific predictions based on *Loripes* and *Dosinia* densities.

The TDRM, which seems to capture the essence of the knots' dietary problem, assumes the following strategy: accept toxic but energy-rich *Loripes* until toxin constraint is met, then add bulky *Dosinia* until the digestive constraint is met. According to our calculations, knots faced both constraints only in 2006, when both prey species occurred in high densities (figure 4*c*; for detailed calculations, see the electronic supplementary material, ‘figure 4*c* explained’). In most years (six of eight; figure 4*c*), however, *Dosinia* was not abundant enough for the birds to become digestively constrained, whereas the presence of *Loripes* was usually high enough to meet the toxin constraint (figure 4*c*). This explains the negative correlation between the *relative* amount of *Loripes* in the diet and the available density of *Dosinia* (figure 5*a*): although the *absolute* rate at which *Loripes* was eaten was likely to be constant each year (equal to toxin constraint *q*), the *absolute* rate at which *Dosinia* was eaten increased with the available *Dosinia* density as long as birds were not digestively constrained (this would occur at a *Dosinia* density of 0.6–0.7 g AFDM_flesh_ m^−2^). A recent study showing year-round changes in *Dosinia* and *Loripes* densities also suggests that the relative contribution of *Loripes* to the diet of knots increased as *Dosinia* stocks became depleted throughout winter [39].

In Banc d'Arguin, knots need an average energy intake rate of approximately 0.2 mg AFDM_flesh_ s^−1^ in order to maintain body mass [67]. In most years, knots would only achieve half of this rate only if they would fully neglect *Loripes* and only accept *Dosinia* as their prey. By adding *Loripes* to their diet, knots would just meet their required energy demand. A plot of the predicted intake rate with (grey band in figure 5*b*) and without (dashed line in figure 5*b*) *Loripes* against the available *Dosinia* densities shows that energy intake rate without accepting *Loripes* would be insufficient for subsistence in 6 of 8 years (also see electronic supplementary material, table S4). Only in 2004 and 2006 would knots have been able to achieve their minimum energetic requirements on *Dosinia* alone (see the electronic supplementary material, table S4; although we modelled knots as ‘intake rate maximizers’, they could just as well have featured as ‘sulphide minimizers’ in these 2 years by fully ignoring *Loripes*; however, the diet data available for 2004 suggest they did not; figure 5*a*).

Note that rate maximization *while* feeding allows for the minimization of *daily* feeding time if a fixed amount of daily energy is required [68]. Minimizing daily feeding time can be beneficial if foraging comes at a cost, such as for example enhanced predation risk [69]. This justifies our approach to analyse survival as a continuous function of intake rate rather than as a simple step function of whether metabolic demands are met. Note further that in poor *Dosinia* years, notably in 2009 (see figure 5*b*; electronic supplementary material, table S4), knots would not even have been able to survive on the combination of *Loripes* and *Dosinia* alone, and would have needed to include other prey types in their diet (which knots indeed did, especially in 2009 [40]).

With *Loripes* and *Dosinia* being by far the most abundant available bivalves at Banc d'Arguin [38], there are not many alternative mollusc prey types to include in the diet. This notion, and the fact that the last years of our study period have not shown high densities of *Dosinia* (figure 4*c*), may explain why the local knot population has declined during especially the second half of our study period (figure 3). However, TDRM energy intake rate showed no trend over time (*r* = 0.51, *F*_1,6_ = 2.07, *p* = 0.20). In addition, also in 1980s, when knot numbers were 40–50 per cent times higher than nowadays [70], *Dosinia* and other non-toxic alternatives were never very abundant [71]. Being a migratory species, it may thus very well be that the carrying capacity of the population is set elsewhere outside Banc d'Arguin [67]; for example, in the Wadden Sea southward staging area, where commercial fisheries led to impaired (re)fuelling opportunities [72].

It is yet unclear what determines the probability of high densities of *Dosinia*, but the negative correlation between annual averages of *Dosinia* and *Loripes* densities is remarkable (figure 4*c*; *r* = −0.76, *F*_1,6_ = 8.30, *p* = 0.03). As has been suggested elsewhere [28], this indicates some form of competition between the two species. Alternatively, there may be differences in environmental conditions among years that steer the negative correlation. For example, observed dynamics in seagrass abundance may underlie this correlation [55,58], with *Loripes* more strongly linked to seagrass habitat than *Dosinia* [38].

It is exciting to hypothesize about how defence strategies in one prey may have been selected for given the defence strategy in another prey. For example, is the bulkiness of *Dosinia* an evolutionary response to the toxicity of *Loripes*? The comparison between the DRM and the TDRM allows us to hypothesize along these lines: it suggests that toxicity of *Loripes* might have increased predation pressure on *Dosinia*, inducing, on an evolutionary time scale, extra armature in *Dosinia*. The reason behind this is that intake rates on *Dosinia* are much higher in the TDRM than in the DRM, especially in years of high *Loripes* abundance (see the electronic supplementary material, table S4). Under the DRM, which treats *Loripes* as if it was non-toxic, knots can reach their digestive constraint on *Loripes* only, leaving no room to add bulky *Dosinia*. By contrast, under the TDRM, many *Dosinia* can be added to the diet because intake rates on *Loripes* are reduced because of the toxicity constraint.

At the same time, the evolution of thick-shelled armature in *Dosinia* may have led to increased predation pressure on *Loripes*, which in turn may have increased *Loripes*'s toxicity. Namely, if *Dosinia* had been relatively thinner shelled than *Loripes* (i.e. when *e_D_*/*k_D_* > *e_L_*/*k_L_*), then knots would prefer *Dosinia* over *Loripes* and would fully neglect *Loripes* in *Dosinia*-rich years. Note that the mechanism of enhanced predation pressure on one prey type as a consequence of induced anti-predator defence in the other prey type proposed here is a classic example of ‘trait-mediated indirect interactions’, which have received renewed attention in the ecological literature [73–75].

With the chemoautotrophically fuelled *Loripes* being the top most abundant bivalve in the system, Banc d'Arguin can be classified as a chemosynthesis-based ecosystem [35]. In contrast to Banc d'Arguin, most chemosynthesis-based ecosystems, such as deep-sea vents and seep systems, are renowned for their lack of predators [19,76,77]. Possibly, such systems lack predators because of the overwhelming densities of toxic prey, whereas non-toxic alternatives are not at hand [78]. The presence of a suitable non-toxic prey may explain why predators are able to thrive at Banc d'Arguin. Hydrothermal vents and deep-sea cold seeps are geographically more isolated than seagrass beds, and also more hostile because of the limited availability of dissolved oxygen in the deep sea. Their isolated positions make it costly for predators to switch between ‘phototrophic’ and ‘chemotrophic’ prey, which could be the reason that such systems are frequented little by predators originating from photosynthetic communities [77,79,80]. By contrast, in seagrass beds, the difference between the anaerobic sulphidic and the aerobic non-toxic environment is just a matter of metres in a horizontal direction (bare versus seagrass mosaics [38,81]), or even centimetres when considered vertically (sulphide concentrations strongly increase in the first 12 cm of the sediment layer [28]). This allows predators to ‘make the best of both worlds’ by adding toxic prey to their non-toxic diet as long as toxin levels do not exceed a given threshold. This mimics the problems recognized long ago for terrestrial herbivores, in which diet selection [82,83], habitat use [84], and fitness and population processes [85] are governed by the occurrence of toxins in the form of secondary plant metabolites or as products from endosymbiotic relationships [86,87]. Our work seems to be the first to make similar problems apparent in a system with predators and prey rather than herbivores and plants.
